# Invasive Cardiopulmonary Exercise Testing in Chronic Thromboembolic Pulmonary Disease; Obesity and the V_E_/VCO_2_ Relationship

**DOI:** 10.3390/jcm13247702

**Published:** 2024-12-17

**Authors:** Estefania Oliveros, Madeline Mauri, Rylie Pietrowicz, Ahmed Sadek, Vladimir Lakhter, Riyaz Bashir, William R. Auger, Anjali Vaidya, Paul R. Forfia

**Affiliations:** 1 Division of Cardiovascular Disease, Department of Medicine, Temple University Hospital, Philadelphia, PA 19140, USAanjali.vaidya@tuhs.temple.edu (A.V.); 2 Department of Medicine, University of California, San Diego, CA 92093, USA; williamrauger@icloud.com

**Keywords:** chronic thromboembolic pulmonary hypertension, cardiopulmonary exercise test, invasive exercise hemodynamics, pulmonary hypertension, right ventricular dysfunction

## Abstract

**Background**: Invasive cardiopulmonary exercise testing (iCPET) provides valuable insight into dyspnea in patients with chronic thromboembolic pulmonary disease, in part through an increased relationship of minute ventilation to CO_2_ production (V_E_/VCO_2_). Obesity lowers the V_E_/VCO_2_ in patients without cardiopulmonary disease; however, whether this holds true in obese subjects with chronic thromboembolic pulmonary hypertension (CTEPH) and chronic thromboembolic pulmonary disease (CTEPD) is unknown. **Objective**: Report on the iCPET findings of patients with CTEPH and CTEPD and investigate the relationship between obesity and gas exchange parameters, especially V_E_/VCO_2_ in these patients. **Methods**: Retrospective analysis of CTEPH and CTEPD patients undergoing iCPET. **Results**: We studied 60 patients; 34 (56.7%) had CTEPH and 26 (43.3%) had CTEPD. The mean age was 61.2 ± 14 years and the mean BMI was 31.8 ± 8.3 mg/kg^2^. A higher V_E_/VCO_2_ (41.9 ± 10.2 vs. 36.8 ± 8.9; *p* = 0.045) was observed in CTEPH vs. CTEPD. There was an inverse relationship between the V_E_/VCO_2_ slope and BMI. For an increase of 1 point in BMI, the V_E_/VCO_2_ slope fell by 0.6 in CTEPD and 0.35 in CTEPH (*p* < 0.001). The mean V_E_/VCO_2_ slope in CTEPH and CTEPD groups was 48.6 ± 10.4 in BMI < 25 and 31.3 ± 6.5 in BMI > 35 (*p* < 0.001). The lower V_E_/VCO_2_ slope in obesity relates to an increased VCO_2_/work rate relationship; there was no difference in the V_E_/work relationship. **Conclusions**: The V_E_/VCO_2_ slope is markedly reduced by obesity, independent of the level of pulmonary vascular obstruction in CTEPH or CTEPD. Thus, obesity masks key physiologic evidence of pulmonary vascular obstruction on the gas exchange assessment of obese individuals.

## 1. Introduction

Chronic thromboembolic pulmonary hypertension (CTEPH) is an uncommon complication of pulmonary embolism (PE) [1]. CTEPH is defined as pulmonary hypertension (PH) with angiographic evidence of organized thrombotic residua within the pulmonary arteries (PA) despite at least 3 months of anticoagulation [2]. For those with imaging evidence of chronic PE in the absence of rest-hemodynamic evidence of PH, the diagnosis of chronic thromboembolic disease, or most recently, the proposed designation, chronic thromboembolic pulmonary disease (CTEPD), is suggested [3,4,5]. Symptoms in patients with CTEPH or CTEPD occur during exercise, yet the hemodynamic assessment is frequently done only at rest. Hence, the importance of invasive cardiopulmonary exercise testing (iCPET), which provides rest and exercise hemodynamic and gas exchange assessment, further defines the physiologic mechanisms of exertional dyspnea. In chronic PE, pulmonary vascular obstruction (PVO) increases the likelihood of exercise-induced PH as well as gas exchange evidence of excessive dead-space ventilation, such as a V_E_/VCO_2_ and a low end-tidal CO_2_ level (PETCO_2_). We report on the iCPET of a cohort of patients with mild-moderate CTEPH and CTEPD. As obesity has been shown to reduce the V_E_/VCO_2_ relationship as compared to non-obese subjects without cardiopulmonary disease, we sought to determine if obesity affects ventilatory inefficiency in patients with chronic PE, in particular the V_E_/VCO_2_ relationship. This investigation assumes greater relevance given the high prevalence of obesity in CTEPD and CTEPH cohorts.

## 2. Methods

### 2.1. Subjects

We conducted a retrospective analysis of 60 patients with CTEPD/CTEPH who underwent clinically indicated iCPET from January 2020 to November 2022. We included adult patients able to exercise with imaging evidence of chronic thromboembolic disease by integrated assessment of results from ventilation/perfusion nuclear scan, computed tomography angiography, and pulmonary angiography. All the patients underwent iCPET. We excluded patients with severe pulmonary hypertension at rest (mPA > 50 mmHg), inability to exercise due to musculoskeletal, acute myocardial infarction (3–5 days), unstable angina, uncontrolled arrhythmias causing symptoms or hemodynamic compromise, acute endocarditis, acute myocarditis or pericarditis, severe aortic stenosis, severe untreated systemic hypertension at rest SBP > 200 mmHg, >120 mmHg diastolic, acute pulmonary embolism. Patients were grouped according to the presence or absence of resting PH into two groups: CTEPH (resting mean pulmonary artery pressure (mPAP) ≥ 25 mmHg) and CTEPD (mPAP < 25 mmHg). These mPAP cutoffs were chosen to maintain consistency between the majority of the published literature on CTEPH and CTEPD where CPET has been applied [6,7,8,9,10] (Supplementary Table S1).

### 2.2. Study Design

iCPET assessment encompasses performing a standard right heart catheterization via the internal jugular vein at rest (supine), followed by a graded exercise on a supine bicycle ergometer with real-time gas exchange assessment and repeat hemodynamic measures. Patients are fitted with a neoprene mask with an aperture for an externally fastened mouthpiece connected via a gas line to a metabolic cart (Ultima CPX™, Saint Paul, MN, USA). Breath-by-breath analysis is obtained, including minute ventilation (V_E_), oxygen consumption (VO_2_), and carbon dioxide production (VCO_2_). Additional variables include power output in watts, ventilatory equivalent for carbon dioxide (V_E_/VCO_2_ slope), PETCO_2_, and O_2_ pulse. The first minute of the bicycle ergometer exercise was performed at 0 watts resistance, followed by a 5-watt per minute ramp in all subjects. Patients were exercised to a goal respiratory exchange ratio (RER) of ≥1.0 and a degree of dyspnea and/or leg fatigue (≥8 on a scale of 0–10). Once patients reached RER and dyspnea thresholds, exercise hemodynamic data were collected. Hemodynamic and physiologic data recorded at rest and relative peak exercise, including systemic blood (BP) pressure by non-invasive cuff assessment, heart rate (HR), pulse oximetry (SpO_2_), mean right atrial pressure (RAP), mean PAP, pulmonary capillary wedge pressure (PCWP), and cardiac output (CO) via Fick equation. The VO_2_ used for Fick CO calculation was directly measured from the metabolic cart simultaneous to obtaining the mixed venous blood sample at peak exercise. Pulmonary vascular resistance (PVR) and systemic vascular resistance (SVR) were calculated in the typical manner. All invasive hemodynamic pressure measurements were obtained at end-expiration and averaged over 10 consecutive cardiac cycles.

### 2.3. Pulmonary Angiography Protocols and the Miller Score Determination

Pulmonary angiography with a digital subtraction system was performed in all patients. Right and left PAs were selectively catheterized, and angiograms were obtained. The investigators scored the PA obstruction using the angiographic index described by Miller et al. [11] The Miller index is a combination of an objective (arterial obstruction) and subjective (peripheral perfusion) score of the lungs. The right PA is assigned nine segmental arteries (3 upper lobes, 2 middle lobes, and 4 lower lobes), whereas the left PA is assigned seven segmental arteries (2 upper lobes, 2 lingula, and 3 lower lobes). The maximal score of obstruction is 16. Reduction of peripheral perfusion is scored by dividing each lung into upper, middle, and lower zones and by using a four-point scale: 0 = normal; 1 = moderately reduced; 2 = severely reduced; 3 = absent. The maximal score of reduced perfusions is 19. Thus, the maximal Miller index is 35 per patient.

### 2.4. Statistical Analysis

Data were analyzed using IBM SPSS Statistics V.22 software (SPSS Inc., Chicago, IL, USA). Descriptive data for continuous variables are presented as mean ± standard deviation or as medians (percentiles 25% and 75%). Categorical data were compared using Fisher’s exact test. Comparisons between groups for continuous variables were performed using unpaired two-sample *t*-tests or the Mann–Whitney test, as appropriate. A sample size of 35 will have 80% power to detect a difference of 0.5 SD in gas exchange parameters using a paired *t*-test with a 1% two-sided significance level. We used the incidence of 2.4% for CTEPH per recent registries for the sample size calculation [1]. Analysis of group effects with repeated exercise measures was performed by comparing mean slope coefficients from individual linear regressions. Pearson correlation coefficients were used to evaluate the univariate relationships between resting and exercise measures of BMI and iCPET parameters. A *p*-value of <0.05 was considered significant. In cases of missing data, albeit minimal, we omitted the missing data and analyzed the remaining data.

## 3. Results

### 3.1. Baseline Characteristics and Comorbidities

Overall, 36 patients (60%) were female, 39 (65%) were White, and 15 (25%) Black. The mean age was 61.2 ± 14 years, and the body mass index (BMI) was 31.8 ± 8.3 mg/kg^2^. The clinical characteristics of the CTEPH (*n* = 34, 57%) and CTEPD (*n* = 26, 43%) groups are presented in Table 1. The CTEPH group was younger than the CTEPD group (57 ± 15 vs. 64 ± 13 years; *p* = 0.05). There were no statistical differences in BMI or comorbidities between the CTEPH and CTEPD groups. Ten (29%) of the CTEPH patients were on PH medications, while none of the CTEPD patients were on PH medical therapy. There was a trend toward a higher Miller Index in the CTEPH group (21.6 ± 4.9 vs. 18.6 ± 7.7; *p* = 0.06).

In Table 2, the results of the rest and exercise studies in patients with CTEPH and CTEPD are presented. The CTEPH cohort had on average mild to moderate PH at rest, a reflection of the less severe PH phenotype that underwent iCPET. No differences in resting HR, BP, SpO_2,_ and 6-min walk distance (6MWD) were observed. Patients with CTEPH expectedly had higher resting mPAP and PVR, as well as higher RAP versus CTEPD. There were no differences in resting CO and cardiac index (CI) between mild-moderate CTEPH and CTEPD.

Echocardiographic parameters were recorded (Table 3). All patients had normal left ventricular function. Seventy-two percent had normal or mild right ventricle (RV) dilation, and 97% had normal or mild RV dysfunction. Sixty-six percent had no evidence of RV outflow tract Doppler notching, supporting a relative lack of excess RV afterload in the overall cohort [12].

### 3.2. Invasive Cardiopulmonary Exercise Test Data

Mean exercise time was 9.4 ± 4.2 min with an average of 53 ± 28 watts attained (Table 2). There was a non-significant trend toward lower exercise time in the CTEPH vs. CTEPD group. There were no differences observed in peak HR and peak systolic BP between groups. In keeping with higher baseline levels of PH, subjects with mild-moderate CTEPH demonstrated higher exercise mPAP, PVR, and RAP than the CTEPD group. Although the PVR was higher at rest and with exercise in the CTEPH group, in both the PVR fell by <10% with exercise. This supports an abnormal pulmonary vascular response to exercise. Despite a higher resting PCWP in CTEPH, there were no significant differences seen in the exercise PCWP between groups nor in the ΔPCWP/ΔCO relationship at peak exercise, with a median ΔPCWP/ΔCO = 1.3 versus 1.8 for CTEPH and CTEPD, respectively. There were no differences in the peak CI achieved during exercise in the CTEPH versus CTEPD groups. However, CTEPH patients exhibited significantly less stroke volume (SV) recruitment at peak exercise as compared to CTEPD; SV index 49.7 ± 18 versus 58.7 ± 18 mL/m^2^; *p* = 0.02.

The RER was similar in the CTEPD (1.03 ± 0.11) and CTEPH (1.00 ± 0.09) groups at peak exercise, suggesting both were at or near anaerobic threshold at peak exercise. The similar RER, nadir SVO_2_ (43.6 ± 10.7 vs. 42.3 ± 9.4%), and peak HR between groups support similar exercise efforts between groups.

There were no significant differences observed in peak VO_2_ or O_2_ pulse between groups. The ΔCO/ΔVO_2_ values between groups were similar, indicating relative preservation of cardiac augmentation in both groups when adjusted for VO_2_.

Similar peak minute ventilation, as well as nadir SpO_2_ values, were observed between CTEPH and CTEPD groups, indicating a relative lack of a ventilatory or pulmonary limitation to exercise in either group. CTEPH patients demonstrated a higher V_E_/VCO_2_ slope, 41.9 ± 10.2 vs. 36.8 ± 8.9 (*p* = 0.045), and lower PETCO_2_ values at rest and peak exercise versus the CTEPD group. These findings suggest that the mild-moderate CTEPH patients possess a greater degree of dead space ventilation than the CTEPD group, in keeping with the trend toward the higher Miller pulmonary arterial obstruction score between groups. Using ROC analysis, the V_E_/VCO_2_ slope had a modest ability to discriminate CTEPH from CTEPD, with a V_E_/VCO_2_ slope of 32.5 (AUC 0.65, *p* = 0.04, CI 0.51–0.79).

We conducted a linear regression model, and the independent variable that affected the V_E_/VCO_2_ slope was BMI (*p* = 0.002). Whereas age, mean PA pressure, PVR, CI, and PA compliance did not show a correlation. Additional models were conducted with comorbidities.

### 3.3. Determinants of Ventilatory Efficiency Based on Body Mass Index

**Figure 1:**
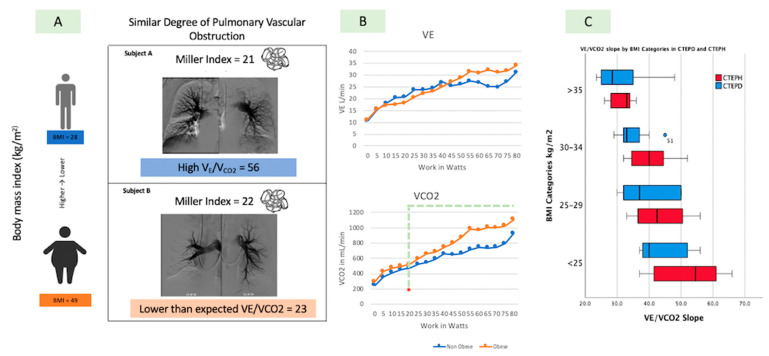
Central Figure. Panel (**A**) Obesity blunts the degree of V_E_/VCO_2_: Similar degrees of pulmonary vascular obstruction have different V_E_/VCO_2_ depending on their BMI (the higher the BMI, the lower than expected the V_E_/VCO_2_). Panel (**B**) Gas exchange parameters in obese (orange) and non-obese individuals (blue). There is no statistical difference in the V_E_ at different work (watts) between obese and non-obese individuals. There are statistical differences (*p* values < 0.05) in the VCO_2_ at different work (watts) that begin at 20 watts (green dotted line and red asterisk) between obese and non-obese individuals. Panel (**C**) V_E_/VCO_2_ slope divided by BMI categories.

**Figure 2:**
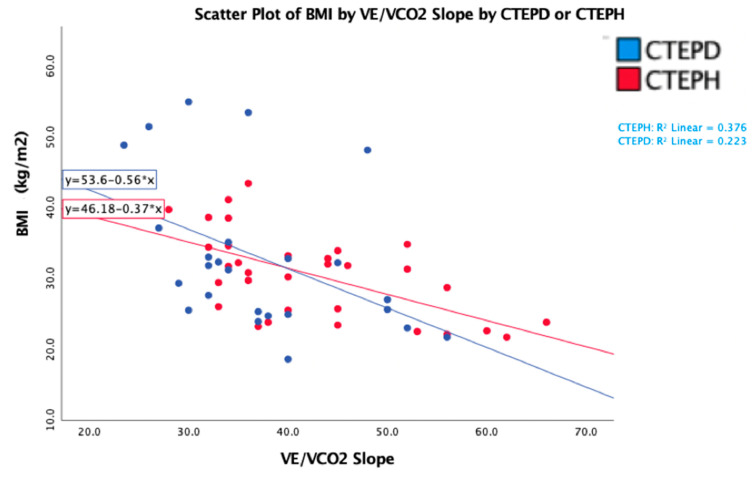
Scatterplot with linear regression of VE/VCO2 slope and BMI in CTEPD and CTEPH.

The V_E_/VCO_2_ slope decreased markedly across quartiles of obesity in both the CTEPH and CTEPD groups (Figure 1 and Supplementary Table S2). The mean V_E_/VCO_2_ slope in CTEPH and CTEPD groups was 48.6 ± 10.4 in subjects with a BMI < 25 and 31.3 ± 6.5 in those with a BMI > 35 (*p* < 0.001). A regression variable plot also demonstrated the negative linear relationship between the V_E_/VCO_2_ slope and BMI (R square of 27%). For an increase of 1 point in BMI, the V_E_/VCO_2_ slope was reduced by 0.6 in CTEPD patients and 0.3 in CTEPH patients (*p* < 0.001). (Figure 2) In contrast to the VE/VCO_2_ slope, PETCO_2_ was similar across BMI quartiles (Table 4).

**Figure 3:**
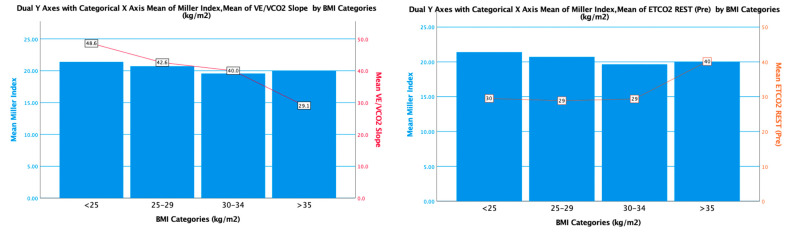
Miller Index by BMI and mean V_E_/VCO_2_ slope and PETCO_2_ at rest in patients with CTEPH and CTEPD. There is a statistical difference between the V_E_/VCO_2_ slope across the BMI categories (*p* < 0.001), the Miller Index is the same amongst BMI categories and PETCO_2_ rest across the BMI categories (*p* < 0.001).

The lower observed V_E_/VCO_2_ slope with increasing BMI could not be explained by a lesser degree of PVO, as there were no differences in the degree of PA obstruction between obese and non-obese individuals (Miller index 20.5 ± 4.8 versus 20.1 ± 7.8, *p* = 0.74 (Figure 3). Therefore, obesity modifies the relationship between minute ventilation and CO_2_ production in the context of comparable levels of thromboembolic disease.

**Figure 4:**
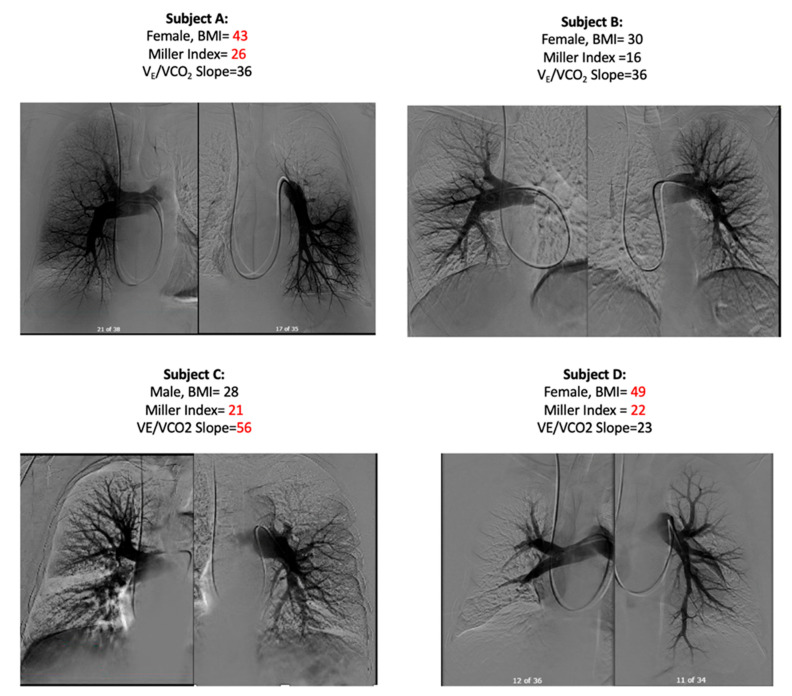
Same V_E_/VCO_2_, but Subject (**A**) has a higher obstruction score and BMI than Subject (**B**). Whereas, Subject (**C**) had a lower BMI and a higher V_E_/VCO_2_ than Subject (**D**), despite having similar obstruction scores.

Representative examples from four subjects are shown in Figure 4. Note how subject A (BMI 43) had a Miller score nearly 1.6 higher than subject B (BMI 30), yet had the same V_E_/VCO_2_ slope. At essentially the same high degree of Miller score, subject D (BMI 49) had a V_E_/VCO_2_ slope half that of subject C (BMI 28).

The lower V_E_/VCO_2_ slope observed in obese subjects was associated with an upward shift in the VCO_2_/work rate relationship versus non-obese subjects. At a workload of ≥20 watts, obese subjects exhibited significantly higher CO_2_ production than non-obese subjects. Similarly, there was a significant upward shift in the VO_2_/work rate relationship in obese versus non-obese subjects. These findings indicate that obese subjects had higher levels of CO_2_ production and O_2_ consumption per level of exercise work. There were no significant differences observed in the V_E_/work rate relationship between obese and non-obese subjects.

The observed differences In V_E_/VCO_2_ between obese and non-obese individuals were not associated with differences in exercise performance or effort. Obese versus non-obese individuals had similar RER values (0.98 ± 0.09 versus 1.01 ± 0.11; *p* = 0.208), exercise time (9.5 ± 4.7 versus 9.3 ± 3.4 min; *p* = 0.09), total watts (49 ± 22 versus 57 ± 34; *p* = 0.288), and nadir PA oxygen saturation (44.8 ± 9.1 versus 40.4 ± 10.6%; *p* = 0.519).

## 4. Discussion

Our study showed CTEPH subjects with relatively mild PH, representing a clinical cohort where we often employ iCPET for further physiologic characterization before deciding on an appropriate intervention such as pulmonary thromboendarterectomy (PTE) or balloon pulmonary angioplasty (BPA). In the context of this relatively mild PH phenotype, the CTEPH subjects had higher rest and exercise RAP, mPAP, and PVR values as well as lesser SV recruitment through exercise as compared to CTEPD. The CTEPH subjects also demonstrated a higher V_E_/VCO_2_ slope compared to CTEPD, in parallel with a trend toward higher PVO scores in the CTEPH cohort. However, the most notable observation in the overall cohort was a strong inverse relationship between the V_E_/VCO_2_ slope and BMI; the V_E_/VCO_2_ slope decreased markedly across quartiles of obesity in both the CTEPH and CTEPD groups. This finding indicates that evidence of ventilatory inefficiency is blunted as the magnitude of obesity increases, which can potentially mask the physiologic evidence of PVO, leading to an underestimation of the role of CTEPD/CTEPH in the functional impairment of obese subjects (Figure 1).

CPET has been used for decades to gain insight into the mechanisms of dyspnea [6,13,14,15,16]. Over the past decade, CPET has been combined with invasive hemodynamic assessment to gain further insight into the exercise physiology of a variety of cardiopulmonary processes, including valvular heart disease, combined pre- and postcapillary PH, and various forms of heart failure [17,18]. Both reduced PETCO_2_ and an elevated V_E_/VCO_2_ are proxies for ventilatory efficiency and, thus, dead space ventilation, which in turn have proven to be reliable physiologic signatures of pulmonary vascular disease across a variety of pH conditions (7–11). Recognizing this, the chronic thromboembolic disease is particularly well suited for iCPET examination, given the cardiopulmonary features of CTEPH and CTEPD often coexist with numerous comorbid conditions, including more advanced age, hypertension, obesity, diabetes, ischemic heart disease, atrial fibrillation, and a propensity for increased left heart filling pressures, which can all contribute to an individual’s functional capacity [19,20,21]. The invasive nature of PTE and BPA interventions places an additional premium on diagnostic precision in chronic PE. Therefore, the incorporation of iCPET as part of chronic PE evaluation when additional physiologic information is needed.

In the current cohort, baseline clinical and physiologic characteristics were similar between CTEPH and CTEPD, including resting HR, systemic BP, oxygen saturation, and 6MWD. These similarities parallel the subtler PH phenotype of the CTEPH patients, with only 3% of the overall cohort having more than mild RV systolic function. In keeping, the resting CI was similar between the mild CTEPH and CTEPD groups, indicating a relative lack of significant RV-PA uncoupling at rest in our CTEPH and CTEPD cohorts (TAPSE/PASP ratio of 0.61 mm/mmHg [normal <0.32 mm/mmHg]) [16,17].

The CTEPH and CTEPD groups put forth similar exercise efforts in terms of RER, peak heart rate, CI, and nadir SVO_2_ at peak exercise. There was an expectedly higher mPAP and PVR at peak exercise in the CTEPH group, in keeping with higher resting mPAP and PVR values in these subjects. In both groups, the PVR did not fall from rest to exercise, characterizing an abnormal pulmonary vascular response to exercise [17,22,23]. Similarly, the change in CO relative to the change in VO_2_ (ΔCO/ΔVO_2_) was similar between the CTEPH (7.8) and CTEPD (7.4), and well within the normal range of 5–8 [24,25,26]. SV augmentation to exercise was significantly blunted, with a 43% increase in SVI observed in CTEPH versus a 74% increase in SV index in CTEPD. These findings parallel those of Claeys et al., who demonstrated that CTEPD subjects demonstrate an intermediary pattern of RV ejection fraction augmentation to exercise, lesser than healthy control subjects but greater than CTEPH patients [7]. This concept seems to apply to CTEPH versus CTEPD whether the PH is mild as seen in our cohort (mean PVR 328 dyne/sec/cm^−5^) or more severe as observed by Claeys et al. (mean PVR 711 dyne/sec/cm^−5^) [7].

In terms of gas exchange data, the CTEPH patients demonstrated a higher V_E_/VCO_2_ slope and lower PETCO_2_ values versus the CTEPD group. These findings suggest that mild-moderate CTEPH patients possess a greater degree of dead space ventilation than the CTEPD subjects. This finding quantitatively and qualitatively parallels prior data indicating a gradation in dead space fraction between CTEPH and CTEPD [7]. However, the gradation in indices of dead space fraction or gas exchange evidence existed in the context of a decidedly milder PH phenotype (mPAP 33 mmHg and PVR 328 dyne/sec/cm^−5^) than in prior studies (mPAP 45 mmHg and PVR 711 dyne/sec/cm^−5^). The differences in V_E_/VCO_2_ and PETCO_2_ in our cohort mirror the trend toward the higher PA obstruction score between CTEPH and CTEPD groups. Prior work has shown that the relationship between V_E_/VCO_2_ and the degree of PVO is dynamic, given the V_E_/VCO_2_ slope has been demonstrated to drop from a baseline value of 50 to 37 post-PTE [5].

Importantly, the strong inverse relationship between V_E_/VCO_2_ slope and BMI occurred despite similar degrees of PA obstruction across BMI quartiles. For an increase of 1 point in BMI, the V_E_/VCO_2_ slope is reduced by 0.35 in CTEPH and 0.6 in CTEPD (*p* < 0.001). In practical terms, these data would indicate that at the same level of PVO, a V_E_/VCO_2_ of 45 in a patient with a BMI of 25 would register as a VE/VCO_2_ of 33 in a patient with CTEPH and 37 in a CTEPD patient if the BMI were 45. We suspect that the stronger impact of obesity on the V_E_/V_CO2_ relationship in obese CTEPD over CTEPH patients reflects a higher baseline physiologic signal for ventilatory inefficiency in the CTEPH subjects, who possess a greater average degree of PVO. Thus, obesity can dramatically mask the gas exchange evidence of pulmonary vascular disease/obstruction, which may lead to the false conclusion that CTEPD or CTEPH are not responsible for a patient’s dyspnea. Our data further indicate that the patient subgroup at greatest risk of dyspnea misclassification is the obese CTEPD patients.

Others have demonstrated an inverse relationship between the V_E_/VCO_2_ slope and levels of obesity in patients without PH [27]. Moreover, the V_E_/VCO_2_ slope increases 3 months following bariatric surgery, indicating there is a dynamic nature to the V_E_/VCO_2_ relationship with obesity [28]. In contrast, exercise PETCO_2_ did not change post-bariatric surgery, a finding that is relatively consistent with the lack of observed differences in exercise PETCO_2_ in relation to BMI in our current cohort. A similar inverse relationship between V_E_/VCO_2_ slope and levels of obesity and BMI has been reported in heart failure with reduced left ventricular ejection fraction and in mixed left heart failure populations [29,30,31].

Although both non-invasive CPET and iCPET have been the focus of several prior studies in CTEPH, the relationship between V_E_/VCO_2_ slope and obesity has not been previously described in CTEPH or CTEPD cohorts. This may relate in part to the fact that these prior studies have focused on non-obese CTEPH populations with a BMI range between 23–28 [5,7,8,10,32]. In contrast, our cohort had a mean BMI of 32, 15% had a BMI over 40, and the highest BMI was 55 kg/m^2^. Thus, the current paper is, to our knowledge, the first to report on the relationship between V_E_/VCO_2_ and obesity in a CTEPH/CTEPD population. These observations may hold particularly important implications in clinical practice given the frequency of obesity in chronic thromboembolic disease overall, as well as the relative importance of V_E_/VCO_2_ as a physiologic marker for severity. More specifically, whether obese subjects with chronic PE and a lower-than-expected V_E_/VCO_2_ relationship are at risk of misdiagnosis and misclassification of their dyspnea to non-CTEPH causes as a consequence of this phenomenon.

The lower V_E_/VCO_2_ slope observed in obese subjects was explained by an upward shift in the VCO_2_/work rate relationship versus non-obese subjects, particularly at higher levels of exercise workload. The lack of observed differences in PETCO_2_ across the quartiles of BMI is consistent with this observation and with prior published data [28].

We also observed markedly higher O_2_ uptake from rest to peak exertion in the obese versus non-obese subjects, which is consistent with the well-known reduction in aerobic work efficiency observed in obese individuals [33]. The observed inverse relationship between the V_E_/VCO_2_ slope and BMI in the current cohort reflects the increased metabolic cost of exercise in the obese.

### Study Limitations

This is a retrospective single-center design with a relatively modest sample size. Our study is, however, the first to investigate detailed rest and exercise physiologic differences between mild CTEPH and CTEPD patients in a carefully phenotyped cohort. We did not exercise individuals with severe CTEPH, which may have introduced selection bias. However, it is typically unnecessary and often unsafe to exercise patients with a severe CTEPH phenotype. The mild nature of the PH in the CTEPH cohort is a potential strength of our study, given that iCPET is more often applied in clinical practice to patients with less severe PH.

Supine exercise may be considered a limitation to the current study or may limit the extension of our findings to non-supine exercise. However, the inverse relationship between V_E_/VCO_2_ and BMI observed in the current study is likely to be further exaggerated during an upright treadmill exercise, given obesity increases metabolic cost through the movement of heavier limbs to a greater extent during weight-bearing exercise such as walking (particularly at an incline) as compared to cycling [34,35,36]. However, although previous studies have shown a higher VO_2_ in upright vs. supine, the V_E_/VCO_2_ slope largely remains unchanged [37]. The interaction of the metabolic cost and the lung mechanics in the different positions is uncontrolled and is a potential limitation. The effect of obesity on End Expiratory Lung Volume (EELV) deserves mention in a protocol using supine ergometry [38,39]. We did not have this data available. Reduced EELV in the supine position can cause lower V_E_ and contribute to a reduced V_E_/VCO_2_ ratio. We studied the effects of obesity on gas exchange parameters in an obese population with an average BMI of 32, where 15% of subjects had a BMI over 40, and we do not have other obesity measurements besides BMI. There may be fundamental differences in the gas exchange alterations of a more severely obese cohort, where the direct mechanical effects of more extreme obesity may lead to relative hypoventilation during exertion.

## 5. Conclusions

The CTEPH subjects also demonstrated a higher V_E_/VCO_2_ slope compared to CTEPD, in parallel with a trend toward higher PVO scores in the CTEPH cohort. However, there is a strong inverse relationship between the V_E_/VCO_2_ slope and BMI, which implies that ventilatory inefficiency is markedly blunted as the magnitude of obesity increases. This can potentially mask the physiologic evidence of PVO, leading to an underestimation or lack of appreciation of the role of chronic PE as the cause of functional impairment of obese subjects.

## Figures and Tables

**Table 1:** Baseline characteristics, comorbidities, and targeted intervention.

Baseline Characteristics	Total *n* = 60 *n* (%) or Mean ± SD	CTEPD *n* = 26 *n* (%) or Mean ± SD	CTEPH *n* = 34 *n* (%) or Mean ± SD	*p* Value	OR (CI)
Age (years)	61 ± 1	64 ± 13	57 ± 15	0.05	
Race/Ethnicity				0.56	
Black	15 (25%)	5 (19%)	10 (29.4%)		
Hispanic/Latinx	5 (8.3%)	3 (11.55)	2 (5.8%)		
White	39 (65%)	18 (69.2%)	21 (61.7%)		
Sex, Female	36 (60%)	19 (73%)	17 (50%)	0.07	0.68 (0.45–1.03)
BMI (mg/kg^2^)	31.8 ± 8.3	33.2 ± 10.5	30.8 ± 6.1	0.27	
Comorbidities					
Asthma or reactive airway disease	5 (8.3%)	2 (7.7%)	3 (8.8%)	0.88	1.14 (0.21–6.37)
Atrial fibrillation or flutter	4 (6.7%)	1 (3.8%)	3 (8.8%)	0.44	2.3 (0.25–20.8)
Autoimmune disorder	10 (16.7%)	6 (23.1%)	4 (11.7%)	0.24	0.51 (0.16–1.6)
Hypertension	27 (45%)	10 (38.5%)	17 (50%)	0.37	1.3 (0.7–2.3)
Chronic Kidney Disease	9 (15%)	2 (7.7%)	7 (20.6%)	0.17	2.68 (0.61–11.83)
COPD	7 (11.7%)	1 (3.8%)	6 (17.6%)	0.09	4.5 (0.6–35.8)
Coronary artery disease	6 (10.34%)	1 (3.8%)	5 (14.7%)	0.44	2.29 (0.25–20.8)
Diabetes Mellitus	10 (16.7%)	5 (19%)	5 (14.7%)	0.45	0.51 (0.16–1.6)
Dyslipidemia	15 (25%)	7 (26.9%)	8 (23.5%)	0.76	0.874 (0.36–2.1)
History of Uterine Fibroids	4 (6.7%)	2 (7.7%)	2 (5.8%)	0.78	0.765 (0.12–5.07)
Hemoglobinopathy	1 (1.7%)	0 (0%)	1 (2.9%)	0.34	0.971 (0.92–1.03)
Hypercoagulable disorder	17 (28.3%)	9 (34.6%)	8 (23.5%)	0.35	1.3 (0.7–2.3)
History of PE	50 (83.3%)	23 (88.5%)	27 (79.4%)	0.35	0.898 (0.72–1.11)
History of Cancer	10 (16.7%)	2 (7.7%)	8 (23.5%)	0.10	3.06 (0.7–13.2)
History of DVT	30 (50%)	12 (46.2%)	18 (52.9%)	0.60	1.147 (0.68–1.93)
Splenectomy	3 (5%)	0 (0%)	3 (8.8%)	0.21	0.94 (0.87 -1.02)
Stroke	4 (6.7%)	4 (15.4%)	0 (0%)	0.02	1.18 (1.0–1.39)
Sleep Disordered Breathing	20 (33.3%)	10 (38.5%)	10 (29.4%)	0.46	0.77 (0.38–1.56)
Tobacco Use History	17 (28.3%)	6 (23.1%)	11 (32.4%)	0.43	1.4 (0.59–3.2)
Thyroid Disorders	8 (13.3%)	4 (15.4%)	4 (11.8%)	0.69	0.765 (0.21–2.77)
Use of PH Medical Therapy				0.73	
PDE5-i	2 (3.45%)	0 (0%)	2 (5.8%)		
Riociguat	6 (10.3%)	0 (0%)	6 (17.6%)		
ERA	1 (1.7%)	0 (0%)	1 (2.9%)		
Prostacyclin	1 (1.7%)	0 (0%)	1 (2.9%)		
Balloon pulmonary angioplasty	13 (21.7%)	4 (15.4%)	9 (26.5%	0.33	
Pulmonary thromboendarterectomy	18 (30%)	6 (23.1%)	12 (35.3%)	0.31	1.5 (0.66–3.53)
Pulmonary Vascular Obstruction Score					
Miller Index	20.5 ± 6.2	18.6 ± 7.7	21.6 ± 4.9	0.06	

Abbreviations: BMI = Body Mass Index; CI = confidence intervals; COPD = Chronic Obstructive Pulmonary Disease; CTEPD = chronic thromboembolic pulmonary disease; CTEPH = Chronic Thromboembolic Pulmonary Hypertension; DVT = deep vein thrombosis; ERA = endothelin receptor agonist; OR = odds ratio; PDE5-i = phosphodiesterase 5 inhibitor; PE = Pulmonary embolism; PH = pulmonary hypertension; SD = standard deviation.

**Table 2:** Baseline hemodynamics and gas exchange parameters.

Baseline		CTEPD (*n*= 26)	CTEPH (*n* = 34)	*p* Value
Watts		57.5 ± 32.8	48.7 ± 23.4	0.24
Time (min)		10.5 ± 4.1	8.6 ± 4.1	0.08
6MWD (m)		372.2 ± 137.2	391.2 ± 114.9	0.62
BSA		1.97 ± 0.29	2.07 ± 0.25	0.40
HR (bpm)	Rest Mean ± SD	79.5 ± 14.1	72.6 ± 13	0.06
	Exercise Mean ± SD	111.9 ± 17.4	107.4 ± 13.5	0.28
SBP (mmHg)	Rest Mean ± SD	135.8 ± 24.9	151.9 ± 20.5	0.98
	Exercise Mean ± SD	135.7 ± 18.2	157.6 ± 38.1	0.19
DBP (mmHg)	Rest Mean ± SD	74.6 ± 14.7	75.6 ± 11.3	0.77
	Exercise Mean ± SD	74.9 ± 13.3	81.7 ± 18.7	0.16
MAP (mmHg)	Rest Mean ± SD	95 ± 16.59	95.7 ± 12.1	0.87
	Exercise Mean ± SD	100.6 ± 12.3	107 ± 23.6	0.02
SpO_2_	Rest Mean ± SD	97.3 ± 7.7	93.6 ± 4.2	0.47
	Exercise Mean ± SD	96.2 ± 3.7	91.7 ± 4.9	0.14
RAP (mmHg)	Rest Mean ± SD	4.8 ± 2.6	8.3 ± 4.4	0.001
	Exercise Mean ± SD	8.5 ± 3.6	14.4 ± 5.8	0.000
Systolic PAP (mmHg)	Rest Mean ± SD	33.2 ± 4.1	53.6 ± 15.1	0.000
	Exercise Mean ± SD	56.5 ± 18.7	82.8 ± 20	0.000
Diastolic PAP (mmHg)	Rest Mean ± SD	13.8 ± 3.5	21.9 ± 6.1	0.000
	Exercise Mean ± SD	23.2 ± 6	33.8 ± 9.4	0.04
Mean PAP (mmHg)	Rest Mean ± SD	20.4 ± 3.1	33.1 ± 8.2	0.000
	Exercise Mean ± SD	34.6 ± 10.3	53.2 ± 14.3	0.000
PA Sat (%)	Rest Mean ± SD	71.8 ± 6.5	66.8 ± 6.2	0.004
	Exercise Mean ± SD	43.6 ± 10.7	42.3 ± 9.4	0.79
PCWP (mmHg)	Rest Mean ± SD	10 ± 3	13 ± 4	0.001
	Exercise Mean ± SD	16.4 ± 6.5	19.3 ± 7.7	0.13
CO (lpm)	Rest Mean ± SD	5.4 ± 1.7	5.1 ±1.2	0.53
	Exercise Mean ± SD	10.2 ± 2.9	9.3 ± 3	0.23
CI (lpm/m^2^)	Rest Mean ± SD	2.7 ± 0.6	2.5 ± 0.4	0.12
	Exercise Mean ± SD	5.1 ± 1.0	4.5 ± 1.4	0.08
SVi (mL/m^2^)	Rest Mean ± SD	34.4 ± 7.4	34.7 ± 6.9	0.47
	Exercise Mean ± SD	58.7 ± 18.2	49.7 ± 18.1	0.02
ΔPCWP/ΔCO		1.55 ± 1.77	1.98 ± 2.02	0.39
PVR (WU)	Rest Mean ± SD	2.3 ± 1.2	4.1 ± 2.3	0.001
	Exercise Mean ± SD	2.1 ± 1.1	3.9 ± 2.2	0.000
SVR (dynes/sec/cm^−5^)	Rest Mean ± SD	1512 ± 483	1536 ± 460	0.86
	Exercise Mean ± SD	739.2 ± 268.8	987.6 ± 504.8	0.08
Pulmonary artery compliance (mL/mmHg)	Rest Mean ± SD	3.7 ± 1.3	2.6 ± 1.1	0.45
	Exercise Mean ± SD	3.5 ± 2	2 ± 1	**0**.003
VO_2_ (L/min)	Rest Mean ± SD	275.56 ± 91.3	301.4 ± 88.4	0.54
	Exercise Mean ± SD	972.92 ± 308	888.75 ± 274.58	0.28
Peak VO_2_ (mLO_2_/kg/min)		10.2 ± 3.7	9.1 ± 3	0.25
O_2_ pulse		11.02 ± 3.7	10.2 ± 2.7	0.44
RER		1.03 ± 0.11	1.00 ± 0.09	0.04
V_E_/VCO_2_ Slope	Mean ± SD	36.8 ± 8.9	41.9 ± 10.22	0.05
PETCO_2_	Rest Mean ± SD	33.77 ± 5.9	30.09 ± 6.78	0.03
	Exercise Mean ± SD	33.28 ± 7.6	27.66 ± 8.27	0.01
V_E_ peaked		33.53 ± 10.10	35.65 ± 11.64	0.49
ΔCO/ΔVO_2_		7.37 ± 3.03	7.81 ± 2.85	0.59

Abbreviations: BSA = Body surface area; CI = cardiac index; CO = cardiac output; CTEPD = chronic thromboembolic pulmonary disease; CTEPH = Chronic Thromboembolic Pulmonary Hypertension; DBP = diastolic blood pressure; HR = heart rate; MAP = mean arterial pressure; Min = minutes; mL = milliliters; PA = pulmonary artery; PAP = pulmonary artery pressure; PVR = pulmonary vascular resistance; RAP = right atrial pressure; RER = respiratory exchange ratio; Sat = saturation; SBP = systolic blood pressure; SpO_2_ = Saturation pulse of oxygen; SVi = stroke volume index; SVR = systemic vascular resistance; V_E_ = minute ventilation; VO_2_ = oxygen consumption; VCO_2_ = carbon dioxide production; WU = Wood Units.

**Table 3:** Baseline Semi-Quantitative Echocardiogram Parameters at the time of CPET.

**Echocardiographic Characteristics**	**All *n* = 60**
Systolic Interventricular Septal Flattening	
None	35 (58.3%)
Mild	25 (41.6%)
Moderate	0 (0%)
Severe	0 (0%)
RV size	
Normal	19 (31.7%)
Mild dilation	24 (40%)
Moderate dilation	17 (28.3%)
Severe dilation	0 (0%)
RV function	
Normal function	36 (60%)
Mild dysfunction	22 (36.7%)
Moderate dysfunction	2 (3.3%)
Severe dysfunction	0 (0%)
RV Shape Base to Apex Ratio	
Normal	44 (73.33%)
Mild	14 (23.33%)
Moderate	2 (3.3%)
Severe	0 (0%)
RVOT Pulse Wave Doppler Notch	
None	40 (66.7%)
Late systolic	7 (11.7%)
Mid systolic	11 (18.3%)
Tricuspid Valve Regurgitation	
None	33 (55%)
Mild	27 (45%)
Moderate	0 (0%)
Severe	0 (0%)
Pericardial Effusion	
None	58 (96.7%)
Mild	2 (3.3%)
Right Atrial Size	
Normal	58 (96.7%)
Enlarged	2 (3.3%)
PASP (Mean ± SD, mmHg)	33.6 ± 13.4
TAPSE (Mean ± SD, cm)	2.04 ± 0.3
LVEF (Mean ± SD, %)	61.2 ± 2.8

Abbreviations: LVEF = left ventricular ejection fraction; PASP = Pulmonary Artery Systolic Pressure; RV = Right ventricle; RVOT = right ventricular outflow tract; SD = standard deviation; TAPSE = tricuspid annular plane systolic excursion.

**Table 4:** Differences in parameters according to body mass index.

BMI (kg/m^2^)	<25	25–29	30–34	≥35	*p* Value
6MWD (meters)	379 ± 170.1	419.9 ± 136.1	391.9 ± 94.9	349 ± 125.4	0.41
Rest PETCO_2_ (mmHg)	29 ± 8	29 ± 5	30 ± 4	38 ± 7	0.21
Exercise PETCO_2_	25 ± 9	28 ± 8	29 ± 6	38 ± 7	0.4
V_E_/VCO_2_ Slope	48.6 ± 10.4	41.4 ± 9.2	38.5 ± 6.7	31.3 ± 6.5	0.09
Rest PCWP (mmHg)	12 ± 4	10 ± 4	11 ± 3	14 ± 5	0.09
Exercise PCWP (mmHg)	15 ± 6	15 ± 6	18 ± 6	23 ± 9	0.36
ΔWedge/ΔCO	1.86 ±2.49	1.03 ± 1.62	1.87± 1.67	2.10 ± 1.87	0.41
Rest PVR (WU)	3.39 ± 1.68	3.74 ± 1.15	3.95 ± 2.63	2.09 ± 1.23	0.36
Exercise PVR (WU)	3.74 ± 2.3	3.59 ± 1.92	3.33 ± 2.05	1.72 ± 1.07	0.27
V_E_ Max	35.1 ± 11.5	37.5 ± 11.2	36.9 ± 12.3	28.6 ± 5.2	0.58
Watts	58 ± 45	60 ± 18	50 ± 23	47 ± 19	0.34
SpO_2_ at the end of exercise in room air	93 ± 6	94 ± 3	92 ± 4	93 ± 5	0.28
FEV1 (%)	95 ± 17	83 ± 20	87 ± 15	80 ± 17	0.25
FVC (%)	90 ± 22	81 ± 14	83 ± 15	78 ± 17	0.79
TLC (%)	78.7 ± 43.4	71.4 ± 56.5	52.3 ± 46.4	46.9 ± 36.3	0.52
DLCO/V_A_ (%)	61.7 ± 22.3	48.8 ± 30.6	86.8 ± 23.7	85.3 ± 22.4	0.43

Abbreviations: BMI = Body mass index; CO = cardiac output; DLCO/V_a_ = diffusion lung capacity of the lungs for carbon dioxide corrected for alveolar volume; PETCO_2_ = partial pressure of end-tidal CO_2_; FEV1 = forced expiratory volume in 1 s; FVC = forced vital capacity; PCWP = pulmonary capillary wedge pressure; PVR = pulmonary vascular resistance; TLC  =  total lung capacity; VA = V_E_ = minute ventilation; VCO_2_ = carbon dioxide production; WU = Wood Units; 6MWDT = 6-min walk distance test.

## Data Availability

The data presented in this study are available on request from the corresponding author.

## Supplementary Materials

The following supporting information can be downloaded at: https://www.mdpi.com/article/10.3390/jcm13247702/s1, Supplementary Table S1. Studies that included CPET in patients with CTEPD and/or CTEPD; Supplementary Table S2. Baseline characteristics, comorbidities, targeted intervention in Obese and non-obese individuals.
