# Supplementary material for: Invasive Cardiopulmonary Exercise Testing in Chronic Thromboembolic Pulmonary Disease; Obesity and the VE/VCO2 Relationship
Source: J Clin Med. 2024 Dec 17;13(24):7702. doi: 10.3390/jcm13247702 (PMC11677292; doi:10.3390/jcm13247702)
Supplement: Supplementary file 1 [file jcm-13-07702-s001.zip › jcm-3368276-supplementary.pdf]

Supplemental Online Table S1. Studies that included CPET in patients with CTEPD and/ or CTEPD

Author (Year)	Mean PAP ranges included
Held (2014)	CTEPH 22-55 mmHg
Swietlik (2019)	CTEPD 14- 25 mmHg
Claeys (2019)	CTEPD 17.5-23.3 mmHg<br>CTEPH 34-51.5 mmHg
Kikuchi (2020)	CTEPH after BPA 19-31 mmHg
Howden (2021)	CTEPH 32-54 mmHg
Ewert (2022)	CTEPH 30- 52 mmHg

Supplemental Table S2. Baseline Characteristics, Comorbidities, Targeted intervention in

Obese and non Obese individuals

Baseline Characteristics	Non Obese N=26 N (%) or Mean ± SD	Obese N=34 N (%) or Mean ± SD	P value
Age (years)	63 ± 16	60 ± 12	0.35
Sex, Female	16 (62%)	20 (59%)	0.8
Asthma or Reactive Airway Disease	0 (0%)	5 (14.7%)	0.04
Atrial fibrillation or flutter	2 (7.7%)	2 (5.9%)	0.7
Autoimmune Disorder	6 (23.1%)	4 (11.7%)	0.24
Hypertension	10 (38.5%)	17 (50%)	0.37
Chronic Kidney Disease	6 (23.1%)	3 (8.8%)	0.12
COPD	5 (19.2%)	2 (5.9%)	0.11
Coronary artery disease	2 (7.7%)	2 (14.7%)	0.78
Diabetes Mellitus	3 (12%)	7 (21%)	0.4
History of PE	23 (88.5%)	27 (79.4%)	0.35
History of Cancer	4 (15%)	6 (17.6%)	0.8
History of DVT	11 (42%)	10 (29%)	0.3
Stroke	1 (3.8%)	3 (0%)	0.4
Sleep Disordered Breathing	5 (19%)	15 (44%)	0.04
Thyroid Disorders	6 (23.1%)	2 (5.9%)	0.052

COPD= Chronic Obstructive Pulmonary Disease; DVT= deep vein thrombosis; PE= pulmonary embolism; SD = standard deviation
